# A Two-Axis Piezoresistive Force Sensing Tool for Microgripping

**DOI:** 10.3390/s21186059

**Published:** 2021-09-09

**Authors:** Bhawnath Tiwari, Margot Billot, Cédric Clévy, Joël Agnus, Emmanuel Piat, Philippe Lutz

**Affiliations:** 1 Department of Automatic Control and Micro-Mechatronic Systems, FEMTO-ST Institute, University Bourgogne Franche-Comté, CNRS, 24 rue Savary, F-25000 Besançon, France; bhawnath.tiwari@femto-st.fr (B.T.); joel.agnus@femto-st.fr (J.A.); emmanuel.piat@ens2m.fr (E.P.); philippe.lutz@femto-st.fr (P.L.); 2 Percipio Robotics, Maison des Microtechniques, 18 rue Alain Savary, F-25000 Besançon, France; margot.billot@percipio-robotics.com

**Keywords:** microrobotics, piezoresistive, multi-axis, design, force sensing, microgripper

## Abstract

Force sensing has always been an important necessity in making decisions for manipulation. It becomes more appealing in the micro-scale context, especially where the surface forces become predominant. In addition, the deformations happening at the very local level are often coupled, and therefore providing multi-axis force sensing capabilities to microgripper becomes an important necessity. The manufacturing of a multi-axis instrumented microgripper comprises several levels of complexity, especially when it comes to the single wafer fabrication of a sensing and actuation mechanism. To address these requirements, in this work, an instrumented two-axis force sensing tool is proposed, which can then be integrated with the appropriate actuators for microgripping. Indeed, based on the task, the gripper design and shape requirements may differ. To cover wide needs, a versatile manufacturing strategy comprising of the separate fabrication of the passive and sensing parts was especially investigated. At the microscale, signal processing brings additional challenges, especially when we are dealing with multi-axis sensing. Therefore, a proper device, with efficient and appropriate systems and signal processing integration, is highly important. To keep these requirements in consideration, a dedicated clean-room based micro-fabrication of the devices and corresponding electronics to effectively process the signals are presented in this work. The fabricated sensing part can be assembled with wide varieties of passive parts to have different sensing tools as well as grippers. This force sensing tool is based upon the piezoresistive principle, and is experimentally demonstrated with a sensing capability up to 9 mN along the two axes with a resolution of 20 *μ*N. The experimental results validate the measurement error within 1%. This work explains the system design, its working principle, FEM analysis, its fabrication and assembly, followed by the experimental validation of its performance. Moreover, the use of the proposed sensing tool for an instrumented gripper was also discussed and demonstrated with a micrograsping and release task.

## 1. Introduction

The requirement for a precise study of the micro world is continuously growing with emerging applications in almost every domain. The manipulation of sub-millimetric size components is notably required in various industrial and scientific applications such as miniature mechanical components [1], optical systems [2,3] assembly, the study of cells (in vivo or in vitro) [4,5], and ultra-small manufactured objects [6,7].

Depending on the specific context of applications and their corresponding precision requirements, the tethered [8,9,10] or untethered [11,12,13] mode of micromanipulation may be employed. With growing needs towards dexterous micromanipulation, the control of contact forces is one of the major issues to tackle. The uncertainty coming from the contact forces may result in losing placement accuracy and also in the damage of the manipulation system (depending on the structure fragility and extent of the contact forces). This gives significant importance to local state knowledge in addressing these challenges. The corresponding state(s) information may be obtained by a mathematical/physical model or by use of the sensor(s). Local states estimation using a model may also be limited by the complexity of environmental inclusion in the model. For example, in [14], an analytical model was used to calculate the strain and curvature, which was then used to estimate the voltage in the piezoelectric material. Such a model was primarily limited by the shape and size of the active material itself; and secondly, by its interaction with electro-mechanical changes from the environment. As a consequence, surface forces have predominant effects in many tasks and are difficult to predict accurately. Measuring these influent forces is required for many applications but for that, adequate sensors, i.e., those that are small enough to enable direct measurement (the closest to contacts), with a sufficient measurement range (typ. *mN*), resolution (typ. sub *μm*) and also having a high dynamic (typ. several hundreds of Hz) [15] is needed.

In the context of micromanipulation, a direct force sensing mechanism [16] can bring more reliant information than curvature sensing alone. Depending on the requirements, several microgrippers integrating force sensing capabilities have been developed in these recent years, such as [17,18]. This capability has been demonstrated as a key interest for the manipulation of biological objects as well as for manufactured microstructures [19]. Moreover, such force sensing enables implementing original robotic strategies that strengthen flexible decision making against an unknown environment [20].

These works demonstrated the key interest of microgrippers with integrated force sensors having suitable performances to successfully achieve microrobotized tasks, however, all of them relied on single-axis force measurement which drastically limits specific tasks’ achievements. Indeed, several works also demonstrate that most tasks at the microscale require considering multi-axis force sensing. This is, for example, the case for the gluing of sub-millimeter scale components [21], the achievement of manipulation tasks with dexterity [22], the characterization of single fibers [23], and the assembly of optical [24] or photonic components [25]. Multi-DoF force sensing such as [26] is thus expected to bring key knowledge for a precise positioning and diverse task handling capability, but the problem of the range of operation and according changes in frames during the tasks can introduce significant limitations to the system. To address this key issue, there is a requirement for an instrumented microgripper capable of multi-DoF force/position sensing which remains an open question.

The microgripper developed in [27] allows a sub-*μ*N force sensing resolution over a range of ±98.27 *μ*N. This device was fabricated with an integrated sensing and actuation mechanism together with one finger instrumented, while the other was dedicated to the actuation purpose. Such a process of manufacturing for the gripper may constitute a high level of fabrication complexity. To address the fabrication complexity, along with the requirement of multi-axis sensing, the adequate force sensing part fabrication can be done separately, which can then be integrated with appropriate actuators to meet the requirements of micromanipulation. This work therefore aimed to fabricate a tethered two-axis force sensing tool. The fabricated tool was then integrated with the appropriate actuator to perform a variety of manipulation tasks at the microscale. The discussion of system design and the sensing principle of the force sensing tool is included in Section 2. Based on the proposed design, an FEM-based analysis is presented in Section 3, covering the expected behavior and performance of the proposed system. The device fabrication, and the assembly process are, respectively, included in Section 4, whereas the experimental demonstration of the sensor performance is presented in Section 5. The proposed force sensing tool is then finally demonstrated with its use as an instrumented gripper achieving the grasping of a micro-object (Section 6), then the final conclusion from the presented work is discussed in Section 7.

## 2. Sensing Principle and Design

In this section, the sensing principle and the design of the force sensing tool are discussed. The device performance we are aiming for in this work constitutes a range of few *μ*N to some mN of the force sensing capability along the two axes, which can be used for instrumented microgripper development. We opted for the piezoresistive sensing principle, as it allows a compatible trade-off in terms of the aimed performances as demonstrated in [16]. The key phenomenon therefore includes the physical change detection (external force) in terms of resistance change and mapping the detected change in terms of force change.

### 2.1. Sensing Principle

A general relation of any conductive structure for the resistance R between the resistivity *ρ*, length L and cross-section area A is defined in Equation (1):

(1) $$R=\rho \frac{L}{A}$$

In order to use any conductive structure as a piezoresistive transducer, the key factors needed are the mechanical reversibility (more particularly, elasticity), a change of electrical resistance and the resistance change detection circuit such as a Wheatstone bridge. The resistance change is not solely dependent on the geometry, or the resistivity, but also on the temperature [28]. Thus, to use any conductive mechanical structure as a piezoresistive transducer, the device must need to have a stable behavior within the operating temperature. This ensures that the significant resistance change comes from the mechanical stress, and not from the small fluctuation in the temperature (or the humidity which is indirectly linked). Therefore, the temperature-dependent element is not included in the definition of resistance in Equation (1). The following discussions in this work will only consider the pure piezoresistive effect, resulting from an external force applied towards the change in electrical resistance. The change of resistance (ΔR) can be written in relation with Poisson’s coefficient ν, the strain ε, and resistivity change Δρ as in Equation (2):

(2) $$\frac{\Delta R}{R}=G\varepsilon \;\mathrm{where}\;G=1+2\nu +\frac{\Delta \rho }{\varepsilon \rho }$$

The term G (Equation (2)) defines the resistance change sensitivity and is called the “Gauge factor”. In [29], the change of Poisson’s ratio (ν) under the strain for different types of material is discussed. In the context of macroscopic isotropic materials, for an extremely compressible material such as foam, ν can go negative up to −1, whereas for a material such as rubber, it can reach max 0.5. Therefore, the dependency on the geometrical parameter to improve the sensitivity is very limited in isotropic materials. For anisotropic materials, although there is no such limits, the possible variation of ν is not very significant [30] and hence has a very limited impact on the gauge factor (G). Therefore, the sensitivity needs to be seen on another dependent factor which is the resistivity of the material. The conventional metal strain sensors typically enable a gauge factor from 2 to 5, but the metal grid strain sensors based on the all-solution process [31] can exhibit a higher gauge factor of 4685.9. A further higher gauge factor can be obtained with the approach of “crack propagation”. Cracks can be introduced in the transverse direction of applied stress, especially in the case of plasma-treated polymers. The introduced cracks lower the conduction and so result in the increase in electrical resistance. Such an approach based on crack depth is discussed in [32], where a very high gauge factor of 16,000 was demonstrated. The use of Pt and Au films in the "crack propagation" approach lacks stretchability (typically <2%). In the context of micromanipulation tasks, the factors such as stretchability, linearity and repeatability are highly important. A high gauge factor exhibiting device may have a very poor stretchability, and therefore both these requirements need to be considered for the sensor development. A semiconductor such as silicon can provide an interesting trade-off between the gauge factor (up to 200) and stretchability (>10% possible) which can be tuned [33] based on the substrate modulus. In the proposed work, a p-type silicon was used for the piezoresistive structure design.

### 2.2. System Design

The piezoresistive sensitivity was defined by the resistance change detection capability. The sensitivity of the device is also dependent on other factors, in addition to the choice of material as discussed in Section 2.1:

(3) $$\frac{\Delta R}{R}=c_{k}G\varepsilon$$

**Figure 1:**
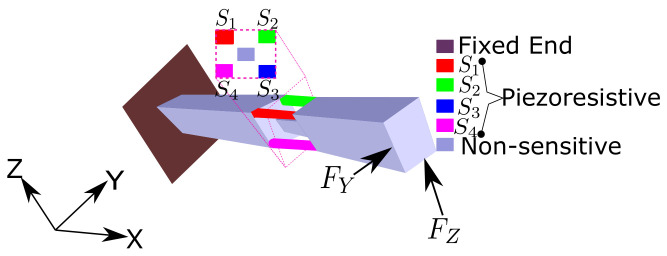
Sensing principle.

The sensitivity of a p-type silicon structure can be further increased (Equation (3)) by the introduction of some cavities. An introduced cavity in [34] made a 25% increase in sensitivity, with $c_{k}$ = 1.25 (scaling factor). Basically, the introduced cavity lowers the mass and so, for the moment of inertia, results in the scaling of the stress distribution in the sensitive part. Therefore, the cavity needs to be taken into account for the system design:

In order to sense forces along the two orthogonal axes, one key important requirement is the distribution of a piezoresistive sensing structure around the neutral axis (leading to a maximization of the stress distribution on the sensitive part). Furthermore, to allow a scaling of the strain distribution, the sensing part needs to be suitably distant from the point of the load and the neutral axis. Moreover, the tool must need to have one end fixed, which is important to have the compression–elongation phenomenon together along the suitable gauges depending on the force applied (discussed in Section 2.3). An overarching schematic, meeting the mentioned requirements, is shown in Figure 1, where four piezoresistive gauges are uniformly distributed around the cavity and the neutral axis of the fixed non-piezoresistive structure.

### 2.3. System Working

**Figure 2:**
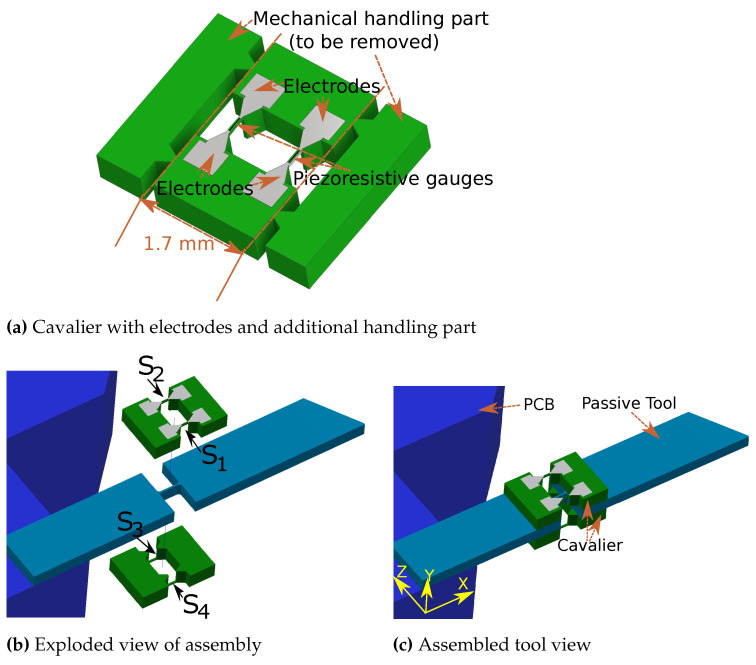
Proposed design of the sensing tool.

The microscale piezoresistive tool needs a clean room-based fabrication process, which mainly includes planar additive or etching processes. The single-wafer fabrication of such a device may introduce complexity in the process, with potential fabrication uncertainties. To overcome the device fabrication complexity, and to target the mass production of a variety of sensing tools with diverse requirements, the related device fabrication may be divided into three parts. In the first part, a mass production of “cavalier” (sensing device containing suspended beams with piezoresistive gauges); secondly, a passive tool depending on specific task requirements can be fabricated separately; in the third part, two cavaliers can be used to assemble onto a passive tool in a way so as to be close to the configuration of Figure 1. With this scheme, the design of a two-axis force sensing piezoresistive tool (PRT) is proposed in Figure 2. The proposed design consists of three parts, a passive tool and two cavaliers which include the piezoresistive strain gauges. The cavalier (Figure 2a) includes additional parts termed “mechanical handling parts”, which can be useful during the process of assembly with the passive tool (detailed in Section 4.3) and later be removed to have the configuration of Figure 2c. Overall, there are five beams passing through the cavity, four beams containing four strain gauges, and one beam is from the passive tool used (exploded view of Figure 2c). The central beam of the passive tool provides a link between the two sides of the cavity (along its length), and is therefore useful to minimize the number of parts and hence the complexity of the development of the sensing tool.

The definition of the strain gauges with respect to the frame defined in Figure 1 remains same for Figure 2; therefore, the top gauges are $S_{1}$ and $S_{2}$, whereas the bottom gauges are $S_{3}$ and $S_{4}$. The corresponding resistances are $R_{1}$, $R_{2}$, $R_{3}$ and $R_{4}$, respectively.

**Figure 3:**
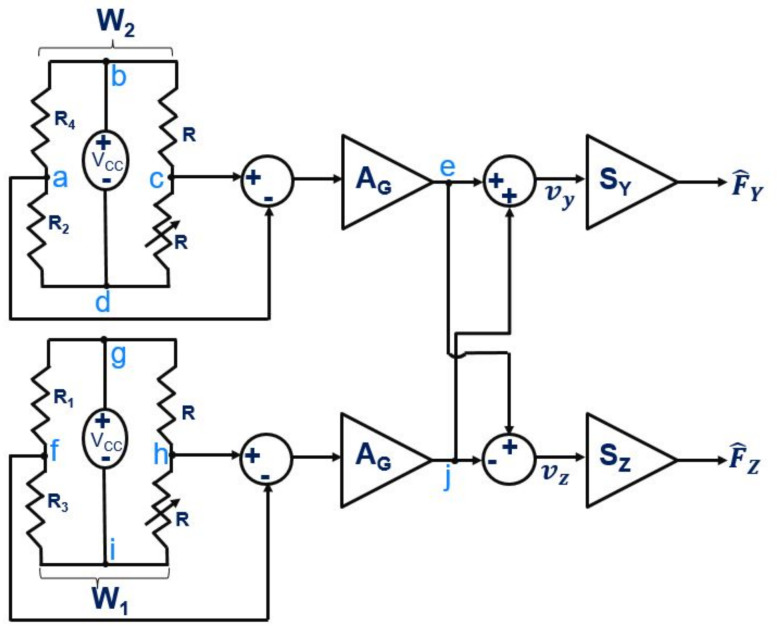
Wheatstone bridges and ${\hat{F}}_{Y}$, ${\hat{F}}_{Z}$ estimation.

Under the applied load along +Y ($F_{Y}$), the gauges $S_{1}$ and $S_{4}$ would be elongated, whereas the gauges $S_{2}$ and $S_{3}$ would be compressed. For a load applied along +Z ($F_{Z}$), the gauges $S_{1}$, $S_{2}$ would be compressed and the remaining $S_{3}$, $S_{4}$ would be elongated (opposite effect with the reversal of direction). This behavior is listed in Table 1, where “+” and “-” are used to indicate, respectively, the elongation and compression of the gauges. Revisiting the obtained behavior along the two axes, it can be seen that the behavior of two gauges remained the same against the load along +Y or +Z. These two gauges are $S_{2}$ and $S_{4}$, where $S_{2}$ undergoes compression for both the cases and $S_{4}$ undergoes elongation in the two respective cases. Furthermore, the other thing that can be observed from the discussed behavior is that the diagonally opposite gauges always kept the opposite behavior irrespective of the applied load in the two cases. These observations can be employed to decouple the force sensing along the two axes. The requirement is to transform the resistance change to force change. This transformation can be achieved with the voltage change detection, which can be done with the help of a “Wheatstone Bridge” circuitry. Using the two diagonally opposite gauges in a Wheatstone bridge, two half bridges can be defined (as shown in Figure 3).

The first bridge $W_{1}$ consists of gauges $S_{1}$ and $S_{3}$, whereas the second bridge $W_{2}$ includes gauges $S_{2}$ and $S_{4}$, each bridge powered with DC supply voltage $V_{CC}$. Each individual bridge is balanced under no-load with two reference resistors R, one of which can be a variable resistor (as denoted in Figure 3) to facilitate the balancing of the bridge against any environmental variation (at no-load). For an input supply voltage $V_{CC}$ in parallel to the bridges $W_{1}$ and $W_{2}$, the respective mid-point voltages of the two bridges at no-load and in balanced condition are, respectively, given by $v_{W1i}$ (voltage at point f and h) and $v_{W2i}$ (voltage at point a and c) (Equation (4)):

(4) $$v_{W1i}=\frac{R_{3}}{R_{1}+R_{3}}V_{CC}\;\mathrm{and}\;v_{W2i}=\frac{R_{2}}{R_{2}+R_{4}}V_{CC}$$

Under the balanced condition, both the middle points of each bridge will have the same potential (voltage at points a and c, and, respectively, at points f and h); therefore, the output of the differential amplifier used (with a gain $A_{G}$) for each bridge would be zero. As a result, the subsequent addition and subtraction of the two output voltages (from points e and j) from the differential amplifiers would also be zero. The respective additions and subtractions are performed to decouple the two axes. The single Wheatstone bridge and the two planar gauges are sufficient to detect the force along Y or Z, but not at the same time. The two bridges, and four gauges distribution are used to detect force along the two axes simultaneously. The detected processed voltage then needs to be multiplied by the sensitivities $S_{Y}$, and $S_{Z}$, respectively, to estimate the force along Y and Z (${\hat{F}}_{Y}$ and ${\hat{F}}_{Z}$). When any load is applied to the sensing tool (in any case, we would be referring to the tool’s tip as the point of load application), then the Wheatstone bridge becomes unbalanced, which would be reflected at a different mid-point voltage (points a, c and f, h) for the two respective arms of each bridge. In reference resistors arms (part of $W_{1}$ and $W_{2}$ where we have R), the mid-point (point c and h) voltages would remain same as that defined for the no-load case, but the arm containing the piezoresistors would have a different mid-point (a and f) voltage following the changes in their respective resistances. Assuming an infinite input impedance of the differential amplifiers used, the current passing through them can be neglected compared to the current passing in the branch; therefore, if Δ$R_{1}$, Δ$R_{2}$, Δ$R_{3}$ and Δ$R_{4}$ are the change in resistance in $R_{1}$, $R_{2}$, $R_{3}$ and $R_{4}$, respectively, then the new mid-point voltages $v_{W1}$, $v_{W2}$ (respectively, in bridge $W_{1}$ and $W_{2}$) can be defined as given in Equation (5):

(5) $$\left\{\begin{array}{c} v_{W1}=\frac{R_{3}+\Delta R_{3}}{R_{1}+R_{3}+\Delta R_{1}+\Delta R_{3}}V_{CC}\\ v_{W2}=\frac{R_{2}+\Delta R_{2}}{R_{2}+R_{4}+\Delta R_{2}+\Delta R_{4}}V_{CC} \end{array}\right.$$

The overall voltage change detected along the Y and Z axes can be given as $v_{y}$ and $v_{z}$, respectively, (Equation (6)), where $A_{G}$ is the amplification gain from the differential amplifier used in the circuitry:

(6) $$\left\{\begin{array}{c} v_{y}=A_{G}\left[\left(v_{W1}-v_{W2}\right)-\left(v_{W1i}-v_{W2i}\right)\right]\\ v_{z}=A_{G}\left[\left(v_{W1}+v_{W2}\right)-\left(v_{W1i}+v_{W2i}\right)\right] \end{array}\right.$$

Combining the corresponding voltage change detected, the amplification and the processing, the force estimated along Y and Z can be written as in Equation (7):

(7) $$\left[\begin{array}{c} {\hat{F}}_{Y}\\ {\hat{F}}_{Z}\\ 1 \end{array}\right]=\left[\begin{array}{ccc} S_{Y}A_{G} & 0 & 0\\ 0 & S_{Z}A_{G} & 0\\ 0 & 0 & 1 \end{array}\right]\left[\begin{array}{ccc} 1 & -1 & -(v_{W1i}-v_{W2i})\\ 1 & 1 & -(v_{W1i}+v_{W2i})\\ 0 & 0 & 1 \end{array}\right]\left[\begin{array}{c} v_{W1}\\ v_{W2}\\ 1 \end{array}\right]$$

Ideally, when there is a force along one axis alone then there should not be any non-zero voltage along the other axis (no coupling), meaning that for $F_{Y}$ alone, $F_{Z}$ should be 0 and vice versa.

## 3. System Analysis in Comsol

**Figure 4:**
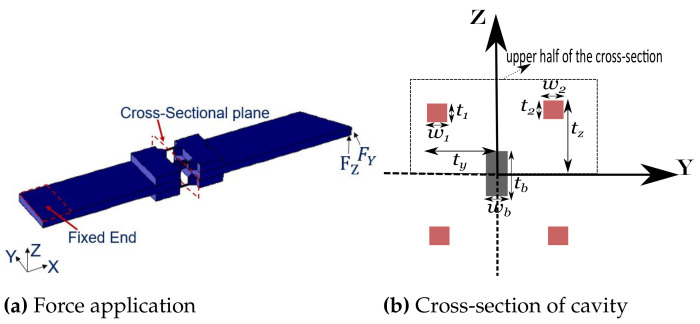
Force application in COMSOL, and the cross-section view of the cavity.

Before getting into the fabrication of the device, it becomes significantly important to estimate the behavior of the aimed sensing tool from the design. We opted for the numerical simulation of the sensing tool presented in the previous section using COMSOL Multiphysics, where the geometrical, physical and electrical parameters are defined in accordance with the standard data-sheet of the corresponding device to be fabricated (key properties listed in Table 2 with geometrical parameters defined in accordance to Figure 4).

In COMSOL Multiphysics, a surface load of 10 mN (across a 50 μm × 50 μm tip area) was applied, respectively, along the Y and Z axes one by one. The stress introduced on the tool was calculated in COMSOL. The sensitivity of the device is a key parameter that can be estimated in COMSOL. In order to estimate that, the stress or strain information is needed. The stress or strain can be directly calculated in COMSOL across a defined region, however, in the experimental scenario, we will not have such a possibility. However, it is possible to develop a model so that the stress or strain can be estimated based on the force applied at the tip. We would cover this discussion in the next subsection.

### 3.1. Modeling of the System for Stress/Strain Estimation

A model used in [35] can enable the estimation of the stress depending on the cavity size. The corresponding estimation was based on the calculation of the moment of inertia (I) and bending moment (M). As the piezoresistive gauges are around the cavity (Figure 4a) and the longitudinal strain is developed along the X axis, the cross-section information of the cavity is therefore needed. A cross-sectional view of the cavity is shown in Figure 4b, a cross-section which is in accordance with the cross-sectional plane marked in Figure 4a. Using the corresponding dimensions listed in Table 2, the moment of inertia along the desired axis can be calculated. Stress is linearly dependent on the *z* which is the distance (where stress is intended to be calculated) from the neutral axis along the direction of the applied load. To simplify the calculation and using the symmetry of the proposed structure (around the cavity, as shown in Figure 2c), only the upper half of the system is sufficient to calculate the stress σ(z):

(8) $$\sigma \left(z\right)=\frac{Mz}{I}\;\mathrm{and}\;I=I_{1}+I_{2}+I_{b}$$

The respective moments of inertia of the gauges $S_{1}$ and $S_{2}$ are denoted as $I_{1}$ and $I_{2}$, and that from the central beam (from passive tool of Figure 2c) as $I_{b}$ in Equation (8):

(9) $$I_{1}=\int_{t-t_{1}}^{t}w_{1}z^{2}dz\;\mathrm{and}\;I_{b}=\int_{0}^{\frac{t_{b}}{2}}w_{b}z^{2}dz$$

A moment of inertia about $S_{1}$ and $S_{2}$ may be assumed equal (symmetric from neutral axis), whereas that along the central beam would be different. The corresponding calculation is shown in Equation (9). The bending moment calculation includes the calculation of the reaction force ($f_{R}$) about the fixed end. Because of the stepped configuration (cavalier-passive tool transition) of the proposed sensing tool, the force applied *F* at the tip of the tool may be approximated to a scaled value for a uniform configuration as proposed in [36]. However, in the present case, because of the complexity from the cavity in the theoretical formulation, a proportional parameter $a_{L}$ to the applied force can be used to define the bending moment at the step near the fixed end. Indeed, it is possible to directly identify a parameter proportional to F in order to estimate M, but keeping an equation of the form Equation (10) allows to have an understanding of the reaction force knowledge which could be useful if the sensing tool is integrated with the actuator or some other passive part:

(10) $$f_{R}=F\frac{x_{L}+a_{L}}{x_{a}}$$

As a result of stepped configuration and the cavity, the reaction force can be defined as in Equation (10). Where $x_{L}$ is the total length of the sensing tool, $x_{a}$ is the distance of the cavalier (nearest edge) from the fixed end, parameter $a_{L}$ can be identified from the least-square fit. Therefore, the overall bending moment at the cavity center can be given by Equation (11), where $x_{B}$ is the distance of the cavity center from the fixed end:

(11) $$M=x_{L}f_{R}-x_{B}F$$

**Figure 5:**
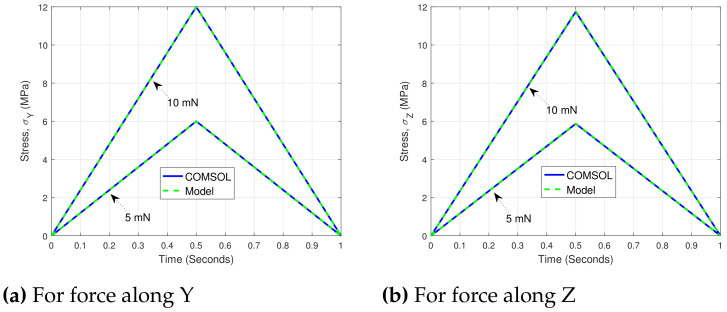
Comparison of analytical model and COMSOL measurement.

For an external force, respectively, *F* = 10 mN and 5 mN (under static conditions), average stress is calculated for $z=t_{z}$ ($z=t_{y}$ for the force along Y). Using the defined model, the corresponding estimate of stress (termed as $\sigma_{Z}$ and $\sigma_{Y}$ for the load along Z and Y, respectively) is compared with the direct measurement from COMSOL and is shown in Figure 5. Because of the simplicity of the model which constitutes the identification of proportional constants $a_{L}$, the entire estimation became much simpler. For parameter identification, MATLAB’s "lsqnonlin" function was used. The corresponding estimation error was less than 5% for all the cases.

For all of the COMSOL simulation results discussed, the physics controlled fine mesh element was used throughout. Calculation corresponding to the load along Y can be performed by the exchange among $t_{1}$ and $w_{1}$, and replacing $t_{z}$ by $t_{y}$ in the defined equations for Z. The identified parameter $a_{L}$ for the load along Z and Y was, respectively, found to be $3.38\times 10^{-3}$ and $4.79\times 10^{-3}$. The identified parameter $a_{L}$ is a proportional parameter and is solely dependent on the geometry of the sensing tool, therefore, for a fixed geometry, it works for any load value within the elastic limit of the structure. One key important side of the stress estimation discussed is its dependency over geometry and the load, whereas the physical material parameters were not needed. This brings the interest of using the discussed model for experimental case, provided the geometrical parameters are the same or in close proximity to what was used in the estimation.

### 3.2. Electrical Connectivity and Gauge Factor Calculation

The interest in this subsection was to combine the electro-mechanical behavior by analyzing the strain change against the resistance change, with which the sensitivity and its according improvement from the cavity can be analyzed:

(12) $$\left[\rho \right]=\rho_{0}\left(1+\left[\pi \right]\left[\sigma \right]\right)$$

To proceed with this analysis, a 5 V DC voltage is applied across the length of the gauge. As there is a change of resistivity ρ under the applied stress (Equation (12)), there is therefore a change in resistance. With the measurement of the resistance change, the gauge factor G can be estimated using Equation (2). Assuming the isotropic distribution of stress, the strain along X and current density was computed in COMSOL. The according resistance can be calculated by knowing the cross-section area of the gauge, the current density and the applied voltage. As the gauge factor is a constant parameter, and therefore irrespective of the load (in the elastic range), the slope of relative change in resistance against the strain should be constant. The slope of this relation was found to be 128.15 and 137.35, respectively, for the load along the Y and Z axes. The $c_{k}$ value obtained (using Equation (3)) is, respectively, 1.05 and 1.125, along Y and Z axes, meaning a 5% and 12.5% sensitivity improvement in the device along the corresponding axes, respectively, due to the cavity.

## 4. Device Fabrication and Assembly Process

In this section, the fabrication and assembly process of the different components of the sensing tool are discussed. The proposed sensing tool consists of two types of components, one being the passive tool and the second being the cavalier with piezoresistive gauges. These two types of components are fabricated separately with two different processes. Their respective fabrication process and assembly is discussed in the following subsections.

### 4.1. Fabrication of the Passive Tool

In this section, the fabrication process of the passive tool is presented.

**Figure 6:**
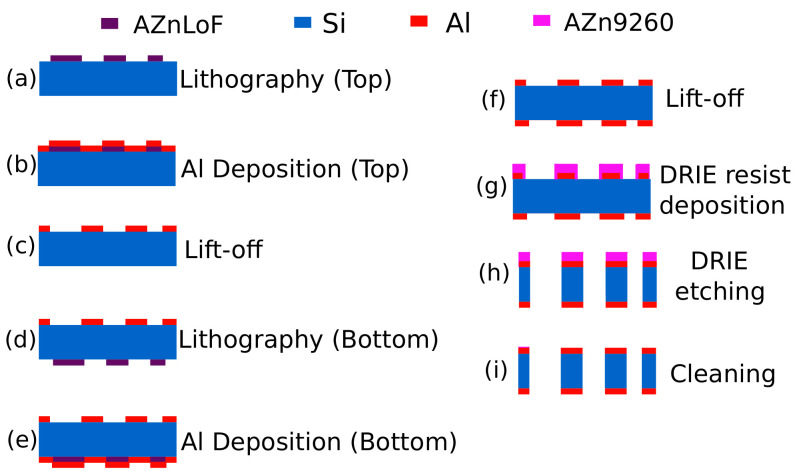
(**a**–**i**) Fabrication steps for the passive tool.

The fabrication starts with a silicon wafer of 350 *μ*m thickness. The top and bottom sides of the wafer went through the lithography followed by the electrode deposition (which may be used for wiring if needed). The DRIE etching technique was used for the etching process. Finally, the samples were cleaned and were ready to be used for the assembly process. A summarized flow of the fabrication process is shown in Figure 6. Both the top and bottom sides used AZ nLOF photoresist for the liftoff of aluminum whereas AZ n9260 photoresist was used for DRIE. Furthermore, before going for the bottom-side Al deposition, the top sides Al was protected by the deposition of C1318 photoresist. Two hundred nanometers of Al deposited on both sides with a deposition speed of 1 nm/sec using a planetary rotation of 5 rpm. For the purpose of lift-off R1165-remover NMP was used. For the alignment of masks and UV exposure, EVG aligner was used with a measured UV power of 9.7 mW and exposure intensity 65 mJ/cm^2^.

### 4.2. Fabrication of the Cavalier

In the presented sensing configuration of Figure 4a, the strain development and the electric current passage for piezoresistive sensing are in the same direction. Therefore, the piezoresistive effect targeted is longitudinal effect. Wafer orientation, type of doping, and the strain direction, can strongly influence the system behavior as discussed in [37]. We chose (100) wafer to have a <110> mobility direction, and therefore, the mask and wafer flat side were accordingly chosen for the fabrication process. The targeted strain development for a piezoresistive effect is longitudinal, and the piezoresitive coefficient for the holes is 718 ${\left(TPa\right)}^{-1}$ which is higher than that of electrons −316 ${\left(TPa\right)}^{-1}$, which makes sense to go ahead for p-doped piezoresistive effect. The n-type wafer can be used to create p–n junctions in reverse bias, so that there is no current leaking into the substrate. This approach of single-crystal diffusion is a widely used method for doping.

**Figure 7:**
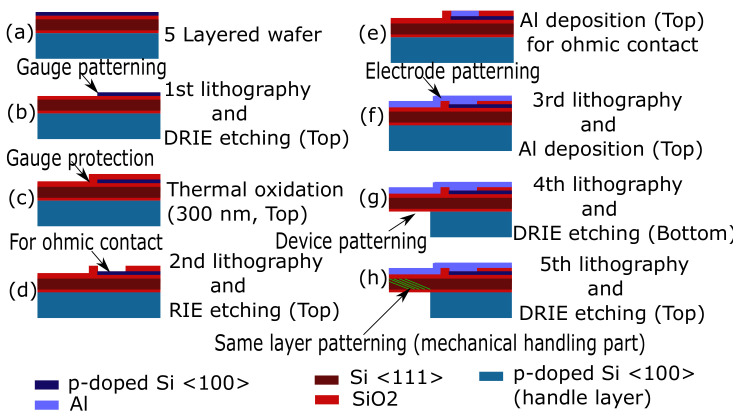
Fabrication process of the cavalier using a 5-layer wafer.

In our case, we used a commercially available 5-layer wafer (5" with 350 μm thickness) which was comprised of one layer of p doped Si <100>, two layers of $SiO_{2}$, one layer of Si <111> and one layer of Si <100> (as shown in Figure 7). The fabrication process (Figure 7) started from the top surface by performing the first lithography followed by DRIE etching. To protect the device layer, the thermal oxidation of 300 nm was then made. The oxidized layered was then partly etched following a second round of lithography on the top surface and the RIE etching. This process was used to create an ohmic contact, which was achieved by etching the oxide and following up Al deposition. A third round of lithography was used on the top side for the deposition of Al to make the conduction around the ohmic contact and to allow the proper routing of electrodes. The fourth lithography was then used on the bottom side followed by DRIE etching for the bottom side. Finally, the last round of lithography was made for the mechanical handling part (side parts of the cavalier as shown in Figure 2a). Similar to the fabrication of the passive tool, the AZ nLOF and AZ n9260 photoresists, respectively, were used for the lift-off and DRIE. The mask alignment machine and exposure intensity and power were found to be the same as what was used for passive tool fabrication.

### 4.3. Assembly Process

**Figure 8:**
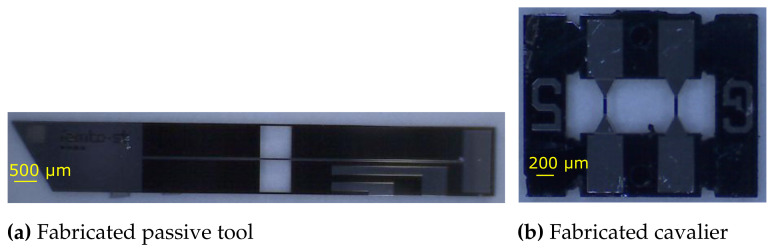
Fabricated parts.

**Figure 9:**
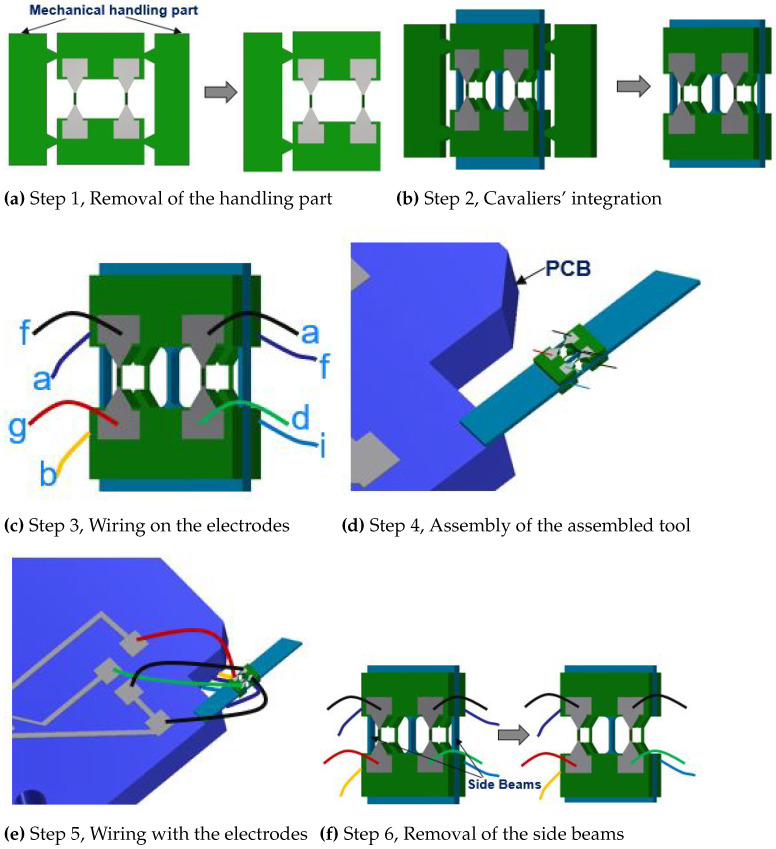
Steps involved in the assembly process.

In this section, the assembly strategy employed in order to develop a sensing tool from the fabricated components was discussed. The fabricated passive tool and cavalier are, respectively, shown in Figure 8a,b. The entire process of assembly was divided into six steps (Figure 9a–f). In step 1, the one side of the mechanical handling part was removed. Thereafter, in step 2, the two cavaliers were glued in a sandwich configuration against the passive tool in such a way that one side only contained one mechanical handling part. This step required extra attention, because the assembly of the cavalier had a direct impact on the behavior of the sensing tool. Therefore, the cavaliers were properly positioned against the passive tool and finally fixed using an epoxy glue.

The remaining mechanical handling parts can then be mechanically removed. In step 3, each electrode around the gauges is electrically wired. The wiring on the electrodes was performed using a conductive glue which needs to be properly cured under the standard condition defined by the manufacturer. The corresponding wiring was made in accordance with the discussed Wheatstone bridge in the Figure 3, where the common point of $R_{2}$ and $R_{4}$ is marked as “a”, and that of $R_{1}$ and $R_{3}$ as “f”. The supply voltage $V_{CC}$ would be provided across the points “b”, “d” and “g”, “i”, respectively, for the bridge $W_{2}$, and $W_{1}$. This assembled tool was then glued on a PCB using an epoxy glue in step 4. After this, the wiring from the electrodes is transferred to the PCB in step 5, which then can be electrically connected to the Wheatstone circuitry as discussed in Figure 3. The extra two-side beams of the passive tool in the cavity part are then removed using a laser cutting process (step 6).

## 5. Experimental Studies

This section introduces the experimental setup and its working and then discusses the realized tasks in terms of system performances. The constituent elements of the experimental setup are defined in Section 5.1.

### 5.1. Experimental Setup

**Figure 10:**
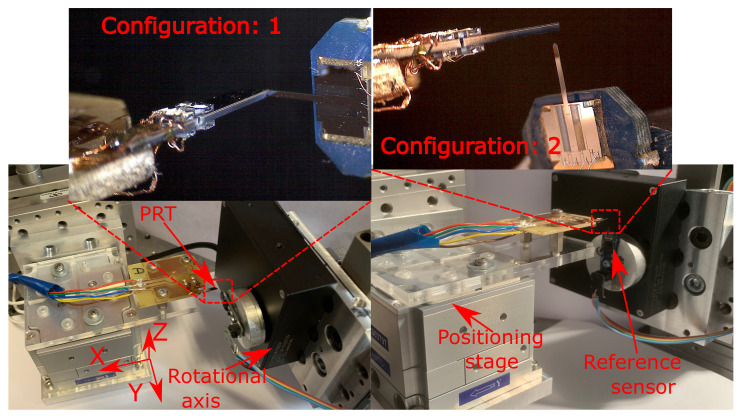
Experimental setup for device characterization (configuration: 1 for the Y axis; configuration: 2 for the Z axis).

The experimental setup of the system is shown in Figure 10. The experimental setup consists of an MCL Nano 3D-200 (termed as positioning stage), which is capable of positioning along three axes with a nanometric resolution. The developed sensing tool was fixed on the positioning stage and was configured to move against a reference force sensor. The reference force sensors employed for the task are FemtoTools FT-S1000 and FT-S10000, respectively, for resolution and long-range validation (as, in our knowledge, there was no commercially available force sensor which can meet both of these requirements). The reference force sensor was fixed on a rotational axis (PI M116.DGH from Physik Instrumente) in order to meet the characterization requirements.

The experimental system configuration for the characterization along the two axes are shown in Figure 10 as configuration 1 and configuration 2 for the Y and Z axes, respectively. Throughout the characterization process, the sensing performances are defined for the applied load within 50 μm from the tip end along the length of sensing tool.

The resistance property is sensitive to environmental variations such as temperature and humidity. In the presented case, experiments are conducted in a closed room assuming negligible influence on the resistance. Depending on the type of doping (p or n) and the doping concentration, the temperature coefficient (a parameter for resistance dependency on the temperature) can change [38]. The resistance across the different gauges under no load is measured (after the wiring and assembly) and listed in Table 3. This corresponding resistance change would lead to voltage changes in the circuitry under the external load. The force sensing PRT can also be used to estimate the displacement of the tip, however, to have this capability, we need to identify the stiffness of the PRT. Such knowledge of stiffness allows one to determine the stiffness of the structure in contact and can also be used for various task handling capacities, for instance, in manipulation against an unknown environment with a dedicated force/position control such as impedance control. For the stiffness identification, the employed experimental setup can be modeled into a spring equivalence system assuming the static behavior of the system. The stiffness of the PRT in general is termed as $k_{PRT}$, whereas the reference force sensor’s stiffness as $k_{ref}$. For a reference force $f_{ref}$, the reference force sensor stiffness $k_{ref}$, and positioning stage displacement along the characterization axis $d_{pos}$, the corresponding stiffness of PRT $k_{PRT}$ can be given as in Equation (13):

(13) $$k_{PRT}=\frac{f_{ref}}{d_{pos}-\frac{f_{ref}}{k_{ref}}}$$

### 5.2. Characterization for Load along Y Axis

**Figure 11:**
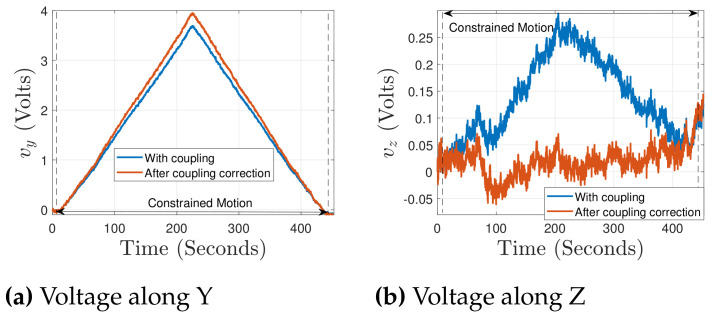
Voltage measured before and after coupling correction, against the external force along Y.

In this section, the different characterization works along the Y axis are discussed. In order to characterize the PRT along this axis, the positioning stage is moved along the Y axis with the displacement $y_{pos}$, resulting in a constrained motion of the PRT against the reference force sensor. The respectively sensed voltages $v_{y}$, $v_{z}$ along the two axes are shown in Figure 11a,b. The applied constrained motion resulted in a major voltage change along the characterization axis, whereas a voltage of approximately 0.3 V along the out-of-motion direction was observed. The alignment of the reference force sensor (sensing axis) and the motion direction (of the positioning stage) was along Y axis and therefore the chances of the force component along Z were significantly reduced. To further minimize the obtained coupling behavior, a scaling factor was identified, which was multiplied by the output of one of the Wheatstone bridge (lower in magnitude), so that the two Wheatstone bridges give the closest possible value. In the present case, a parameter $k_{v}$ equal to 1.15 was identified.

**Figure 12:**
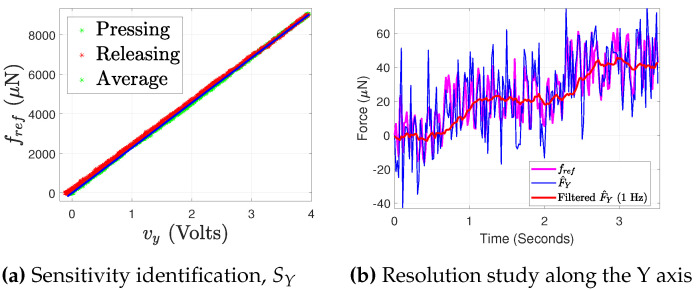
Characterization of PRT along Y.

The voltages obtained after coupling correction along Y and Z are, respectively, shown in Figure 11a,b, where the coupling extent is typically minimized to the noise level in the signal processed. From here onward, the respective voltage changes detection and the related calculations along the two axes would be considered after the coupling correction. The employed Wheatstone bridge circuitry allows the measurement in terms of voltage under the application of any external load, and therefore there is a need to find a factor which can map the voltage measured in terms of force. This factor is the sensitivity of the proposed PRT. The force measurement from the reference force sensor ($f_{ref}$) against the sensed voltage ($v_{y}$) along the Y axis is shown in Figure 12a. An estimate of force is calculated using an identified sensitivity of 2280 *μ*N/V. The force measurement of 9 mN is presented in Figure 12a. For the PRT tip position under a constrained motion, the stiffness of the proposed sensing needs to be calculated. For this, using Equation (13) with the stiffness of the PRT along Y axis $k_{YPRT}$, the corresponding positioning stage displacement $y_{pos}$ is substituted in the place of $d_{pos}$, and a $k_{ref}$ equal to 8300 N/m (for the FT-S10000) is used. This resulted in the stiffness $k_{YPRT}$ of PRT along the Y axis as 5130.3 N/m. The sensing resolution of the force sensor can be defined in terms of the percentage of the noise level obtained from the acquisition of the signal, but it would be interesting to validate the system capability to detect the minimum change experimentally. In order to demonstrate the sensing resolution, FT-S1000 is used as the reference force sensor.

A staircase motion of the 30 nm step was made from the positioning stage along the Y axis. Under the constrained motion, the respective sensed force from the reference force sensor and the sensed force from the PRT is shown in Figure 12b. The proposed PRT is able to detect the force change of 20 *μ*N corresponding to the detection from the referenced force sensor.

### 5.3. Characterization for Load along Z Axis

**Figure 13:**
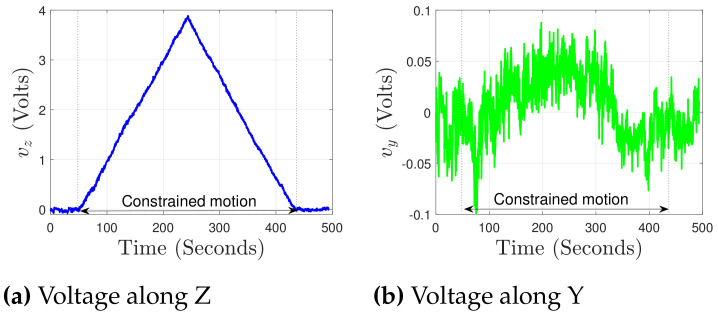
Characterization of PRT along Z.

**Figure 14:**
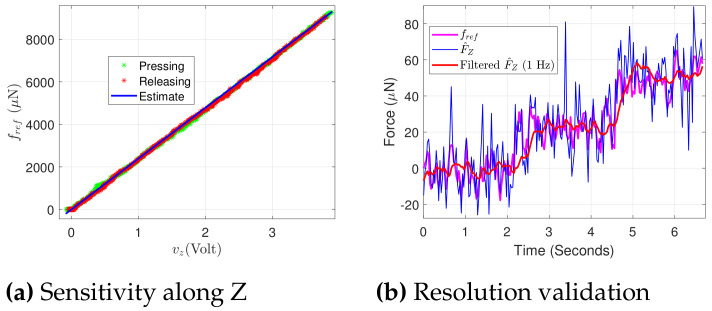
Sensitivity identification and resolution validation under external force along Z.

The characterization along the Z axis of the sensing tool was performed using configuration 2 of Figure 10. The same process was followed as the one performed for the Y axis. The corresponding reference force and the voltages (Figure 13a,b) are shown in the Figure 14. The validated force ranging along the Z axis is 9.2 mN (Figure 14a) with 20 *μ*N of resolution (Figure 14b).

## 6. Proposed Sensing Tool for Instrumented Microgripper Development

The proposed PRT was demonstrated with a two-axis force sensing capability with performances as listed in Table 4. In this section, the discussed PRT was used to develop an instrumented microgripper. For the development of an instrumented but multi-axis microgripper from the developed PRT, the primary requirement was to integrate the sensing tool with the precise multi-axis actuators (for multi DoF tasks). The actuation of any microgripper can be chosen from the different existing principles such as electrostatic [39], electromagnetic [40,41], pneumatic [42], electrothermal [43], vacuum [44], shape memory alloy [45] or piezoelectric [46]. One important point in the choice of the actuator is that the actuator should not interfere with the performance of the sensing device integrated; therefore, in the context of the piezoresistive tool for sensing, it is important to avoid external stimuli such as heating or optical-based actuation. The requirement is also to avoid the need for any high input voltage (or additional amplifier), by keeping a precise and long-range positioning capability.

**Figure 15:**
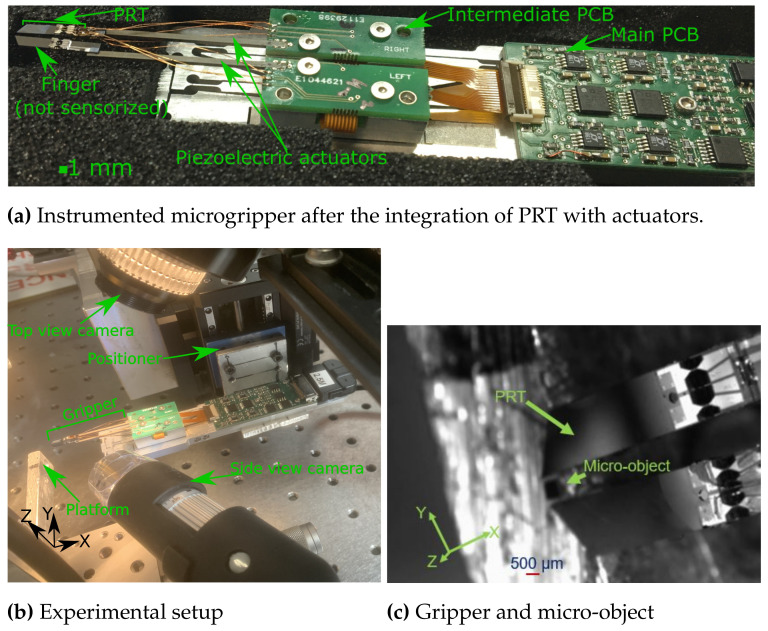
Instrumented microgripper and experimental setup.

The opening of the gripper sometimes requires the generation of additional forces to get rid of contact forces, and therefore the actuation needs to be compatible along both sides (±) of the respective axes. Piezoelectric actuators are known for their precise positioning, and have the advantage of no electromagnetic radiation, a good response time with a simple structure and are widely used in precise positioning applications [47,48,49]. In this work, the piezoelectric multi-layer actuators are used for the development of the gripper. This piezoelectric multi-layer actuators can be actuated with a nanometric resolution up to ±100 μm (range) along the two axes, corresponding to a maximum input voltage of ±20 Volts. Indeed, a bimorph actuator can introduce a higher blocking force compared to a multilayer stack piezoelectric actuator. However, in our case, the sensing range of the integrated PRT and the according task handling force requirements are well within the blocking force of the employed piezoelectric actuator (>10 mN). The choice of piezoelectric multilayer actuator meets the compatibility with the developed sensing PRT and also the requirement of micro-manipulation. The PRT presented in the previous sections is integrated with two-axis piezoelectric actuators and the assembled version is shown in Figure 15a. The electronics including the circuitry for actuation and PRT, which consists of Wheatstone bridges (included in the “main PCB”), are developed on board with the gripper, where the “intermediate PCB” is mainly used to allow a smooth transfer (closest to PRT, low noise) of electrical signals to the “main PCB”.

**Figure 16:**
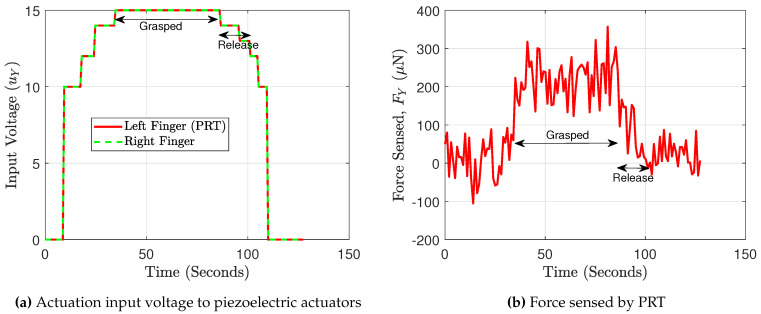
Grasping and release of a micro-object.

The proposed gripper consists of a single finger instrumented (marked as a PRT in Figure 15a) for sensing while the other finger is not wired for piezoresistive sensing and therefore only used for the actuation/gripping purpose. The piezoelectric actuators are fixed on an intermediate PCB and the electric signals are then processed to the main PCB before the exchange of the signals with the host PC. The grasping and the releasing of a micro-object (size of 500 × 500 × 350 μm^3^ made of silicon, fabricated in a clean room) was demonstrated using the developed instrumented microgripper. The experimental setup to perform the grasping/release was shown in Figure 15, where the PI M-122.2DD positioning stages were used (marked as “positioner”). With the help of these positioning stages, the gripper was well positioned around the micro-object placed on the platform (as shown in Figure 15c). Using the cameras (top view and side view camera as shown in Figure 15b), it is ensured that the gripper is well positioned relative to the micro-object to grasp. Following which, a step-wise input voltage is supplied to the actuators (symmetrically) to follow a gripping motion (along the Y axis) while monitoring the relative distance between the gripper and the object using the camera. The corresponding force needed to grasp the object should be very much less based on its weight but the object in general may constitute significant surface forces because of the interaction with the substrate upon which the object is placed. Therefore, to be sure about safe grasping, a little extra force of 200 *μ*N was applied. The grasp of the micro-object, is verified by making a motion of the gripper in the Z direction (using the positioner). Finally, the gripper was allowed to move closer to the manipulation platform and the actuator of the gripper was allowed to then move step-wise to release the micro-object on the platform. The duration for which the micro-object was grasped, is marked as “Grasped” and that during the process of release as “Release” in Figure 16a,b. With the proposed instrumented microgripper, it is possible to safely manipulate micro components.

## 7. Conclusions

The proposed work introduced a novel multi-axis force sensing tool, which works on a piezoresistive principle. The corresponding sensing principle, design and working scheme, FEM analysis, device fabrication and assembly were discussed. A force sensing resolution of 20 *μ*N over a range of 9 mN was experimentally demonstrated along the two axes. The proposed sensing tool was further demonstrated as an instrumented microgripper for an application that consists of grasping a micro-object. As a future work, the proposed instrumented microgripper can be used to handle complex micro-assembly tasks such as gluing, rotation, and insertion. Moreover, it would be interesting to make an adjustment in the device material choice, partly so as to have a variable stiffness microgripper. A lower stiffness version of the device could also be useful for the manipulation of many tiny biological components. These additional interesting advancements would be the goals of future works.

## Figures and Tables

**Table 1:** External force influence with direction.

Force	$S_{1}$	$S_{2}$	$S_{3}$	$S_{4}$
$F_{Y}$	+	−	−	+
$F_{Z}$	−	−	+	+

**Table 2:** Geometrical and mechanical parameters used in the COMSOL simulation.

Parameters	Value
$t_{1}$, $t_{2}$ (μm)	40
$w_{1}$, $w_{2}$ (μm)	50
$w_{b}$ (μm)	50
$t_{b}$ (μm)	350
$t_{z}$ (μm)	575
$t_{y}$ (μm)	525
Poisson’s Ratio, ν	0.27
Piezoresistive Coefficient, π (${\left[TPa\right]}^{-1}$)	718
Young Modulus, E (GPa)	170
No load Resistivity, $\rho_{0}$ (Ωmm)	0.25

**Table 3:** Resistance of gauges under no load.

Resistors	Values (kΩ)
$R_{1}$	2.98
$R_{2}$	3.01
$R_{3}$	3.22
$R_{4}$	2.88

**Table 4:** PRT performance summary.

Parameters	Y	Z
Stiffness (N/m)	5130.3	2342.4
Sensitivity (μN/V)	2280	2390
Resolution (μN)	20	20
Standard Deviation (μN)	28	22

## Data Availability

The data presented in this study are available on request from the first or the corresponding authors.
